# Canine-Assisted Interventions in Hospitals: Best Practices for Maximizing Human and Canine Safety

**DOI:** 10.3389/fvets.2021.615730

**Published:** 2021-03-30

**Authors:** Sandra B. Barker, Nancy R. Gee

**Affiliations:** Center for Human–Animal Interaction, School of Medicine, Virginia Commonwealth University, Richmond, VA, United States

**Keywords:** animal-assisted intervention, best practices, human–animal interaction, canine-assisted intervention, human–canine interaction

## Abstract

Canine-assisted interventions (CAI) are becoming more popular in hospital settings, representing a crucial intersection between animals, veterinary medicine, and society. However, standardized policies and procedures to minimize risk and maximize benefit to vulnerable humans and protect therapy dog welfare are lacking, posing a challenge to safe practice. Few intervention programs are evaluated to document efficacy compounding the potential risk. This paper presents a rationale for CAI in hospitals and describes the evidence, issues, and challenges to establishing and maintaining safe and effective programs for humans and animals. Recommendations are made for best practices based on the existing scientific evidence and a model program in place in a major medical center for 19 years. Scientific and practical implications are considered.

## Introduction

Hospitalized patients represent a vulnerable population as they face not only the challenges of their medical conditions and treatments, but also separation from familiar settings and social supports. Recognizing the distress associated with hospitalization, administrators, and clinicians seek novel, complementary, and cost-effective interventions to provide comfort and support to patients. Perhaps in part due to broad media claims of patient benefits, canine-assisted interventions (CAI) are becoming more popular in hospital settings. However, both a lack of consistent practice standards and evidence of program efficacy pose risks to patients, hospital employees, therapy dogs, and dog handlers. This paper describes the evidence, issues, and challenges for CAI programs to practice safely and effectively in hospital settings and provides recommendations for best practice based on scientific evidence and a model program.

## Evidence of Canine-Assisted Intervention Efficacy

### Benefits of Human–Animal Interaction

Humans have shared their lives with companion animals for thousands of years, yet it has only been over the past three to four decades that researchers have turned their attention to the possible health benefits of this relationship (1). The health outcomes and populations studied are widely varied and although there are some promising findings, overall, results have been mixed. The areas with the most substantial evidence supporting health benefits of human–animal interaction are cardiovascular disease risk and stress reactivity. Research on cardiovascular benefits of pet ownership was launched by a 1980 seminal study documenting decreased mortality in pet owners 1 year after discharge from a coronary care unit (2). Further research supported these findings in a sample of pet owners participating in the large Cardiac Arrhythmia Suppression Trial (3). Social support ant pet (particularly dog) ownership predicted 1-year survival for survivors of myocardial infarction, independent of demographics, disease severity, and other psychosocial factors. Decreased cardiovascular disease death was also reported in a longitudinal study of a nationally representative sample of pet owners without major physical illness (4). In this study cat ownership was significantly associated with reduced risk of death, particularly from stokes.

Other studies documenting reduced cardiovascular reactivity in pet owners followed. Married couples owning dogs or cats had lower baseline diastolic and systolic blood pressure than couples without pets and showed lower reactivity and faster recovery to mental and physical stressors (5). Lower cardiovascular reactivity to mental stress was also found in adults with borderline hypertension randomly assigned to obtain a cat or dog and start ACE inhibitor therapy compared with those on angiotensin-converting enzyme inhibitor therapy only (6). Systolic blood pressure, heart rate, and renin activity were significantly lower in the pet owning group. Lower physiological stress has also been reported in military veterans with post-traumatic stress disorder living with service dogs (7).

Lower systolic and diastolic blood pressure were found in older adults with pre- to mild hypertension when their pet dogs were present during their normal daily lives (8). Similarly, a large Australian study of cardiovascular risk factors in pet owners and non-owners attending a free medical screening reported pet owners had lower systolic blood pressure and triglycerides, and exercised more (9). More recently researchers analyzed data from 24-h heart rate monitoring of pet and non-pet owners and determined that pet ownership served as an independent modulator of cardiac autonomic imbalance in patients with diabetes mellitus, hypertension, and hyperlipidemia (10).

After a critical review of studies such as these, an American Heart Association scientific statement was published concluding that “pet ownership, particularly dog ownership, is probably associated with reduced cardiovascular risk” and “pet ownership, particularly dog ownership, may have some causal role in reducing cardiovascular disease risk” [Levine et al. (11), p. 2356].

### Benefits of Canine-Assisted Interventions in Hospitals

A natural extension of this body of work is to examine whether humans in settings without dogs, such as hospitals, can benefit from safely interacting with unfamiliar dogs, as is the case when interacting with visiting therapy dogs. An exploratory study compared physiological benefits of dog owners interacting with their own therapy dog vs. an unfamiliar therapy dog in a clinic setting (12). This small study examined patterns of physiological reactivity (salivary cortisol, systolic and diastolic blood pressure, heart rate, brain waves) to a mental stressor and found consistent patterns of increased stress associated with the stressor and consistent patterns of relaxation associated with interacting with a therapy dog in both conditions. Relaxation patterns observed interacting with an unfamiliar therapy dog were consistent with those observed when interacting with one's own dog and self-reported anxiety and stress were similar in both conditions, providing preliminary support for further study of CAI in healthcare settings.

Although studies have emerged supporting the benefits of CAI in hospital settings, little consistency exists in the clinical populations studied, outcomes, and methodologies emphasizing the need for more studies in this area. Inpatient psychiatry is one area with more consistent evidence of patient benefits. An early study of the effect of CAI on anxiety in hospitalized psychiatry patients yielded promising findings. Patients with mood, cognitive, psychotic, or other disorders had lower anxiety scores on the State-Trait Anxiety Inventory after participating in recreational therapy incorporating a CAI (13). Although between group differences were not significant, only patients with mood disorders had lower anxiety scores in the comparison group receiving traditional recreational therapy without a dog present. Similar findings were reported in a crossover study of acutely depressed patients assigned to CAI and a control condition (14). State-Trait Anxiety Inventory scores were lower in the CAI condition.

Other studies reporting CAI benefits for hospitalized psychiatric patients found reductions in fear prior to electroconvulsive therapy in patients randomly assigned to a 15-min CAI vs. magazines (15), greater reductions in depression, anxiety, pain, and pulse in patients receiving CAI compared to a comparison stress management program (16), qualitative references by adolescent patients to a CAI therapy dog as a friend or therapist and increased interactions on the psychiatric unit promoted by a CAI (17), and enriched therapeutic contact and enhanced patient openness and treatment adherence associated with CAI on a psychiatric service (18). Two studies reported increased attendance by psychiatry patients in group therapies involving CAI compared to group therapies without CAI (19, 20). Several studies have focused on benefits of CAI specifically for inpatients with schizophrenia. Improvements in negative symptomatology, greater treatment adherence, and reduction in cortisol levels were reported in patients randomly assigned to psychosocial rehabilitation including CAI (21). Benefits were also found for patients randomly assigned to weekly CAI for 2 months with improvements in self-esteem, self-determination, and positive psychiatric symptoms (22). Hospitalized elderly patients with schizophrenia were the focus of another study, reporting improvements in socialization, well-being, and activities of daily living following 12 months of weekly CAI (23).

Although several studies have investigated the effects of CAI on pediatric patients, results have been mixed with two randomized controlled studies reporting no effect on biobehavioral stress (24), or self-reported anxiety or pain (25). Similar null findings were found in a study of physiological stress, anxiety, and medical fear in pediatric patients assigned to CAI or a comparison condition (26). In contrast, other studies have reported positive findings, including reductions in anxiety in a convenience sample of pediatric patients assigned to CAI compared to a control group (27), greater reductions in physiological and behavioral distress in children undergoing a physical exam with a dog present compared with those in a control group (28), and less distress and lower cortisol levels in pediatric patients when participating in CAI before, during, and after venipuncture compared with a control group (29). As with dog ownership studies, varying methodologies, techniques for sampling biomarkers, samples and sample sizes, and target outcomes likely contribute to the mixed findings seen in CAI studies. Advances in technology and assessment methods informing later studies likely contribute to differences as well.

Other hospitalized patient populations have been the focus of one or two studies of CAI indicating potentially positive effects, but further study is needed with these populations to confirm results. For example:

Patients randomly assigned to CAI prior to cystoscopy had lower anxiety and stress levels than a treatment as usual control group (30)
Patients randomly assigned to physical therapy incorporating CAI following total joint arthroplasty reported less pain and higher satisfaction with their hospitalization than those assigned to standard physical therapy (31).
Following total joint replacement surgery, patients from two hospitals were compared in a retrospective study of matched samples. Patients participating in CAI used less oral pain medication than the control group (32).
Patients with moderate or greater anxiety in an emergency room had lower anxiety following CAI than those in a control group (33).
Patients with heart failure selected to walk with a dog had a lower ambulation refusal rate (7.2 vs. 28%) and achieved greater ambulatory distance than a historical sample not participating in CAI (34).
Cancer patients participating in CAI during chemotherapy had greater reductions in depression and increased arterial oxygen saturation compared with a control group (35).

In addition to the benefits of CAI reported for patients, hospital healthcare professionals were also found to benefit from CAI in a pilot study documenting reduced physiological stress. Significant reductions in both serum and salivary cortisol were detected 45 min after 5 and 20 min of CAI (36). No cortisol differences were found between either treatment condition and 20 min of quiet rest suggesting that hospital staff may benefit from very brief CAI.

The promising evidence of CAI benefits for various hospital populations coupled with the low-cost of CAI programs utilizing community volunteer therapy dog teams support CAI as a feasible complementary intervention to traditional medicine. However, the vulnerability of hospital patients and unique environment of the hospital setting pose challenges to conducting CAI safely and effectively for patients, staff, dogs, and volunteers.

## Issues and Challenges

**Figure 1:**
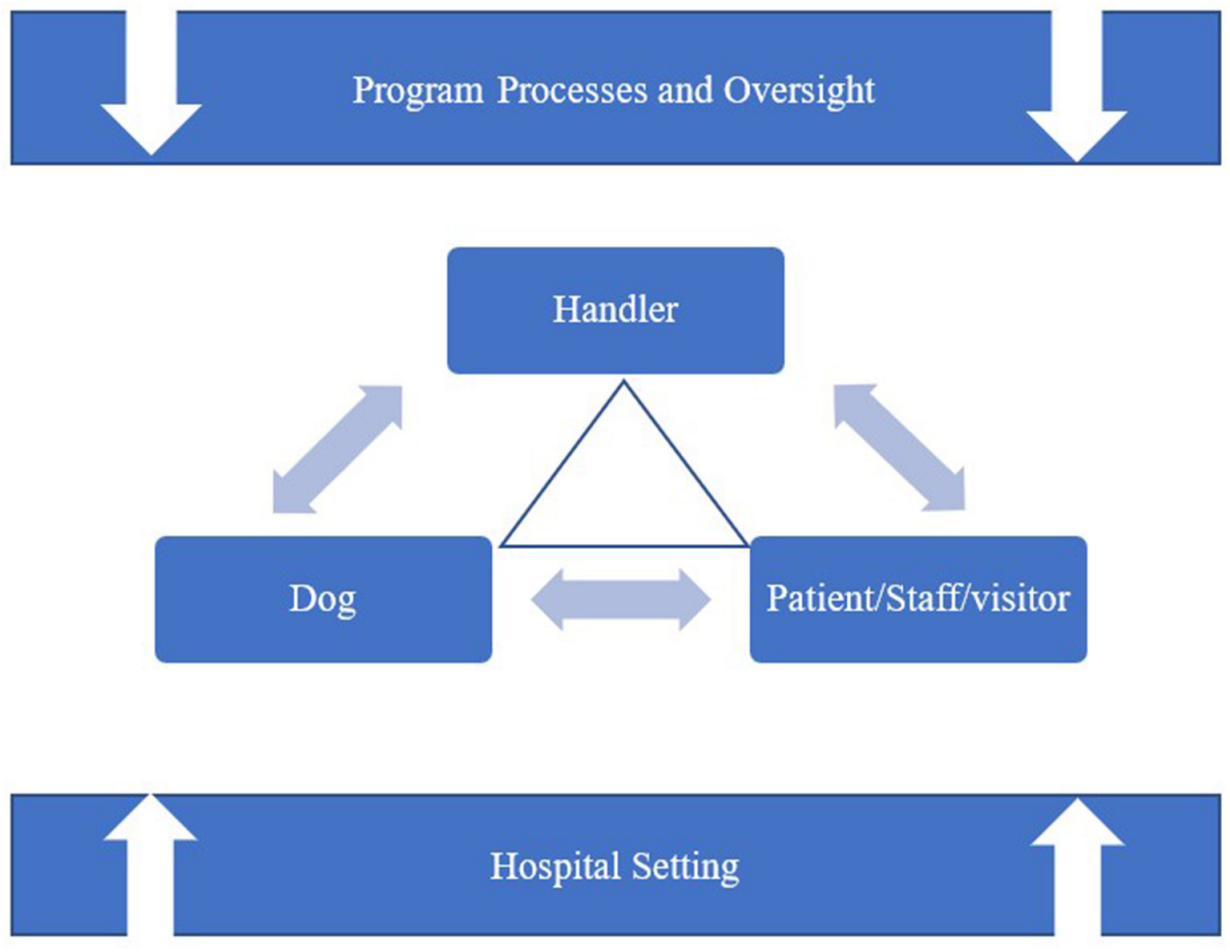
Sources of potential issues and challenges.

Therapy dog visitation programs in hospitals present many potential issues and challenges stemming from multiple sources. Figure 1 presents a depiction of how the hospital setting and the accompanying program processes and oversight may impact the handler-dog-patient (staff member or visitor) triad.

### Hospital Setting

Hospitalization is stressful for patients and their families (37) and evidence suggests that staff may suffer from compassion fatigue or burnout which places patients at risk from errors, abuse, or neglect (38) and has the potential to exacerbate an already stressful environment. For example, stressed nurses are more likely to make errors, less likely to interact with patients (helping them to cope with their illness) or each other (creating an isolated and competitive work environment) (39). The hospital setting also tends to include stressors that impinge upon human and animal senses. Visually, hospitals may be crowded, with people moving about quickly, or include seriously injured or sick people who may move unexpectedly or with great urgency. Hospitals may be loud with irregular sound interruptions from overhead paging systems, beeping machines, or patients expressing pain or discomfort. There may be wide variety of strong odors including antiseptic or cleaning fluids, vomit, or burnt flesh. Surfaces in hospitals tend to be smooth, hard, or flat for easy cleanup or built for utility rather than comfort so the tactile experience is also not particularly relaxing. This variety of sensory experiences can be stressful and likely impacts on all three points of the triangle (e.g., dog, handler, patient) depicted in Figure 1.

Humans, including patients, staff, and visitors, may experience a variety of psychological and physiological stress reactions that are both acute and chronic in nature (40). Well-trained and temperament tested dogs who are newly placed into a hospital setting may become stressed and less responsive to their handlers. We will focus our discussion on each of the three aspects of the handler/dog/patient triad, but it is important to remember that many of these issues or challenges overlap with, and apply to, more than a single aspect on the triad.

### Handler Concerns

Handlers are faced with a variety of potential challenges in hospital settings including routine practical issues such as what items to bring with them, where to park their car or exercise their dog, to more complex issues like how to safely avoid exposure to infectious diseases, and what topics of conversation are appropriate with each patient or visitor. In most, but not all cases, the dog handler is a person who is volunteering their time to bring their own dog to the hospital for visits. The handler has typically invested a sizeable amount of time and energy into training, testing, and registering their dog with an appropriate therapy dog organization (e.g., Pet Partners). Research on human–dog attachment has shown that attachment styles tend to mirror those found in mother–child relationships (41) and similarly influence stress coping, such that secure attachment tends to be associated with better stress coping. This may suggest that handlers with secure attachments to their dogs may more effectively cope with the wide variety of stressors in the situation.

Canine-assisted interventions (CAI) handlers often train their dogs to be in close proximity to, and interact with, strangers which appears to create behavioral adaptations during CAI, such that the dog may maintain greater eye contact with their handler as a way of maintaining contact during uncertainty (42). Attachment styles and behaviors indicative of maintaining contact during CAI are taken as additional indicators of the strength of the bond between the human and the animal. It is important to consider this bond as both a positive and a negative aspect of CAI because it is likely to influence handler behaviors and reactions during CAI. On the positive side, the bond between handler and dog may drive the handler to pay close attention to all things that may affect, or stress, their dog and act immediately to ensure the safety and well-being of their dog. It is critical to the safe practice of CAI to have a person in the environment whose role it is to focus exclusively on the health and well-being of the animal. They should be vigilant for things that might harm the dog, and for signs of stress or discomfort in the dog and act accordingly to protect the safety of the animal. On the negative side, a strong bond between handler and dog may contribute to handlers using poor judgment regarding their dog's behavior in the environment. For example, they may allow the dog to perform behaviors (e.g., certain tricks or off-leash walking) that the dog may enjoy, overlooking how safe or appropriate those things may be in that setting.

If a handler notices that their dog is stressed, their role is to remove the animal from the situation, but they are frequently confronted with opposing pressures to extend the visit. For example, the handler may see that a person is enjoying interacting with the dog, or the person may be in pain (emotional or physical) and taking great comfort from the dog, or the person may be alone and near the end of life and may simply want to touch the dog for a little longer. Handlers frequently struggle with what they may perceive as the morality of ending the visit under these more extreme circumstances. For this reason, it is important to prepare handlers for these challenging situations, give them tools to help them to deal with each individual situation appropriately, and provide ongoing support. Furthermore, there is some evidence to indicate that owners may not be well-prepared to recognize signs of stress in their dogs, and that they may benefit from educational efforts to improve their ability to recognize and interpret signs of stress in their own dog (43).

Like hospital personnel, handlers are subject to the stresses inherent in a hospital environment. Exposure to patients who are suffering, facing terminal illness, and who die during hospitalization may contribute to handler anxiety and stress and secondary traumatization. Handlers who regularly visit a service may see a patient several times and 1 day finds the patient gone. Since they do not have access to confidential patient information, handlers are left wondering about the status of the patient. Unlike hospital personnel who can debrief with colleagues about patient conditions, including the death of a patient, and access employee assistance programs, handlers are left to process such situations alone unless support services are made available to them. Compassion fatigue is a risk for handlers that may lead to burnout, withdrawal from volunteering, or compromise their ability to safely provide CAI (20).

### Canine Concerns

In an attempt to establish standards of welfare for livestock animals, the Brambell Report defined ideal states known as the Five *Freedoms* (44). These are freedom from (1) hunger and thirst, (2) discomfort, (3) pain, injury, or disease, (4) fear and distress, and (5) the freedom to express normal behaviors. The Five Freedoms have been widely accepted and adopted by many professional organizations (e.g., the American Veterinary Medical Association) across a wide array of research and applied settings. Sandoe and Christiansen (45) built on these fundamentals by attempting to define what might constitute a good animal life in which it is important to consider that animals have needs and preferences that may be different from those of humans. Another concept relevant to the inclusion of animals in human–animal interaction is that of *a life worth living* (46). This idea addresses the animal holistically over the course of their lifetime and is based on basic states, overall welfare, value of life, and quality of life. Related to the concepts of the Five Freedoms is the Five Domain Model which has been updated and revised over the past 20 years to incorporate scientific thinking into the assessment of animal welfare [for a review see (47)]. This model considers both negative and positive affective states or experiences, and explores concepts such as agency of the animals involved in human–animal interaction research and practice.

Because it is a human choice to include the dog in animal-assisted interventions, it is incumbent upon the humans to make sure that the dog is experiencing a life worth living, and the Five Freedoms provide a starting point for this discussion. When a dog enters a hospital setting it is critical that the dog is consistently well fed and hydrated, free from disease and parasites, calm, unstressed, and demonstrating behaviors indicative of comfort with people and the surrounding environment. The primary responsibility for ensuring these things falls to the handler, in collaboration with their veterinarian (for the dog's health and wellness), the therapy dog registering agency (e.g., Pet Partners, Alliance of Therapy Dogs), and local program administrators and evaluators.

A 2017 review of the existing evidence (*N* = 17 studies) indicated that the use of aversive training methods jeopardizes both the physical and mental health of dogs (48). A recent empirical investigation comparing positive (i.e., beneficial) methods to the use of a shock collar provided further evidence to support these conclusions. The study demonstrated that dogs trained with positive training techniques achieved better responses and shorter latencies to common commands (e.g., “sit” and “come”) than the dogs in the aversive training condition (49). The authors conclude that positive training was more effective and posed fewer risks to dog welfare and the quality of the human–dog relationship.

Routine veterinary wellness exams, vaccinations, and fecal exams to check for internal parasites help handlers to monitor the health of their dogs and reduce the risk of zoonotic disease transmission between humans and dogs (e.g., rabies). The recent COVID-19 pandemic has highlighted the fact that it is not possible to completely eliminate the risk of disease transmission (e.g., a novel virus) among humans and dogs, but monitoring the ongoing health of both species can reduce the risk and allow for early detection and treatment of potential health issues.

Safe dog–human interactions require the understanding of dogs' signaling behaviors and there is a striking lack of knowledge of these behaviors in the general public (50). Shepherd (51) proposed a ladder of dog behaviors indicating escalating levels of distress. On the lower steps in the ladder the dog will demonstrate several appeasement and calming behaviors such as yawning and nose licking, turning their head or body away, and walking away. As they feel more stressed, they may pin their ears back, tuck their tail under their body, stand in a crouch, lying down, leg up. As they become distressed, the dog may stiffen their body and stare, growl, snap, and finally bite. It is critically important to the welfare of the dog, for handlers and program personnel to be able to identify low level signs of stress and to act immediately to remove the animal from the situation. Acting upon this knowledge can defuse a situation, make interactions more enjoyable for the dog, and create an environment that is more relaxed, enjoyable, and respectful of the unique contributions of the dogs.

There are many potential risks to dogs in a hospital setting, requiring the handler to be vigilant in monitoring their dog's safety. For example, something may have been dropped on the floor (e.g., medication) of a patient room or spilled (e.g., body fluids) in the hallway. Equipment can be both fragile and top heavy, so if it is inadvertently knocked over the dog may become injured. Equipment and people can block or crowd a pathway, creating obstacles that may be intimidating to the dog, requiring the handler to recognize and carefully navigate unexpected situations.

Patients can also be a source of risk to the dog. Some patients may have difficulty with gross or fine motor movements and may stumble onto the dog, or roughly grab at the dog. Other patients can become overly interactive with the dog and hug them tightly or attempt to pick them up or move them in ways the dog does not enjoy. Some patients will attempt to share their food or drinks with the dog. Children may pull their hair or tail, and I.V. bags and lines may startle the dog if they become entangled in them.

The handler carries a heavy responsibility in monitoring their dog, their interactions with humans, and any potential risks in the environment. It is important for the CAI program and hospital to provide education and support to handlers in carrying out these responsibilities.

### Patient/Visitor/Staff Concerns

Patient and healthcare provider safety is a top priority for all healthcare facilities, including hospitals. Therapy dog presence represents a risk to that safety in the form of potential zoonotic pathogen transmission, cross-transmission of human pathogens, injury, and symptom exacerbation.

Insufficient research has been conducted investigating the role of dogs as a vector for zoonotic disease transmission in hospitals (52). We were able to find no studies investigating therapy dogs and cross-transmission of human pathogens. One study examined hospital infection rate as a pilot CAI program was being implemented in an Italian pediatric hospital (53). The hospital's infection control committee independently monitored infection rates over a 12-month period. Although attendance at the weekly CAI was high, no increase in infection rate compared with the previous year was found.

Two recent surveys conducted in the United States examined CAI health and safety policies and practices. One study surveyed 45 hospitals, 45 senior care facilities, and 27 therapy dog organizations with results showing inconsistent policies to safeguard patient health and animal welfare (54). Particularly alarming, the survey found 70% of therapy dog organizations surveyed permitted dogs to be fed a raw meat diet. A nationally representative survey of U.S. therapy dog organization practices reported similar results of inconsistent practices that may risk human health and canine welfare (55). Results also revealed a concerning number of organizations failing to limit raw meat diets and treats and failing to place time limits on therapy dog visits.

Feeding a raw meat diet remains a contentious subject among some therapy dog owners. A study of salmonella and other potential pathogen risk in therapy dogs in Canada fed a raw meat diet found dogs fed raw meat at some point during the yearlong study were more likely to test positive for salmonella and extended-spectrum cephalosporinase than dogs not fed raw meat (56). No differences were found in associations between eating raw meat and *Clostridioides difficile*, methicillin-resistant *Staphylococcus aureus*, or vancomycin-resistant Enterococci.

In the absence of studies providing more conclusive evidence on the role of canines in zoonotic pathogen transmission and cross-transmission of human pathogens, strict infection prevention and control procedures, including the ability to contact trace CAI interactions, must be established for CAI to maximize patient, healthcare provider, and CAI team safety. Two recent publications address this issue and provide guidelines for the safe practice of CAI in hospitals (52, 57).

In addition to zoonotic disease transmission, therapy dogs represent a risk for injury to patients, visitors, and healthcare personnel as well as to other therapy dogs if providing CAI with other therapy dog teams. Although evaluated for health and temperament by therapy dog organizations to minimize such risk, dogs still have the potentially to trip, scratch, or bite someone in the environment or accidently interfere with equipment (e.g., knocking over equipment). Hospital policies and procedures must address such risks, provide direction for handling such incidents, consider liability issues, and develop procedures to minimize risks.

Dogs in the environment also present a concern for patients, visitors, and staff with allergies to canine dander and fear of dogs. CAI programs must address these potential risks to maximize human safety.

An additional potential issue for handlers, patients, and hospital personnel is the illness and/or death of a therapy dog. For the handler, serious illness or death of their therapy dog often represents a significant loss (58, 59). Not only have they lost a beloved canine family member, but unless they own more than one therapy dog, they also lose an important activity and the relationships and gratification associated with being a hospital volunteer and providing CAI. Processes are needed to support handlers at such times. Patients and personnel routinely visited by a therapy dog may also be saddened and grieve when a therapy dog becomes seriously ill or dies (60). Patient and staff support services need to be cognizant of the significance such incidents may pose and provide appropriate resources.

A less obvious issue for handlers and patients is the ability of therapy dog teams to meet the demand for CAI. Patients and staff requesting CAI may be disappointed if the visit does not materialize. This can be particularly difficult for pediatric patients who may be informed that a therapy dog will be coming or observe the CAI team visiting other patients and leaving the unit without seeing them. Staff training in understanding the limits of CAI resources and presenting the intervention as requested but not guaranteed can help minimize negative reactions when a requested visit cannot be fulfilled.

For CAI to be successful, hospital staff must understand basic information about the approved CAI program. Staff must be informed of areas approved for CAI, criteria for determining patient appropriateness to participate, and procedures to request CAI for their patients. An understanding of therapy dog and handler requirements to deliver CAI is important to maximize appropriateness of requests. Ideally key hospital administrators and staff are involved in the development and ongoing evaluation of any CAI program in their setting.

## Recommendations for Best Practice

Best practice is defined by Merriam-Webster (61) as “a procedure that has been shown by research and experience to produce optimal results and that is established or proposed as a standard suitable for widespread adoption.” Therefore, to be suitable for widespread hospital adoption, a CAI best practice program must be evidence-based and historically shown to produce recognized high-quality results with minimal negative effects. Such a program should be time-tested, address the canine, handler, patient, visitor, and staff concerns described in the previous sections, and be shown to be effective in the hospital setting. We present best practice recommendations with examples from a program model that meets these best practice criteria. The inclusion of experiential information related to the model program is unavoidably subjective.

### Program Model: Dogs on Call

**Figure 2:**
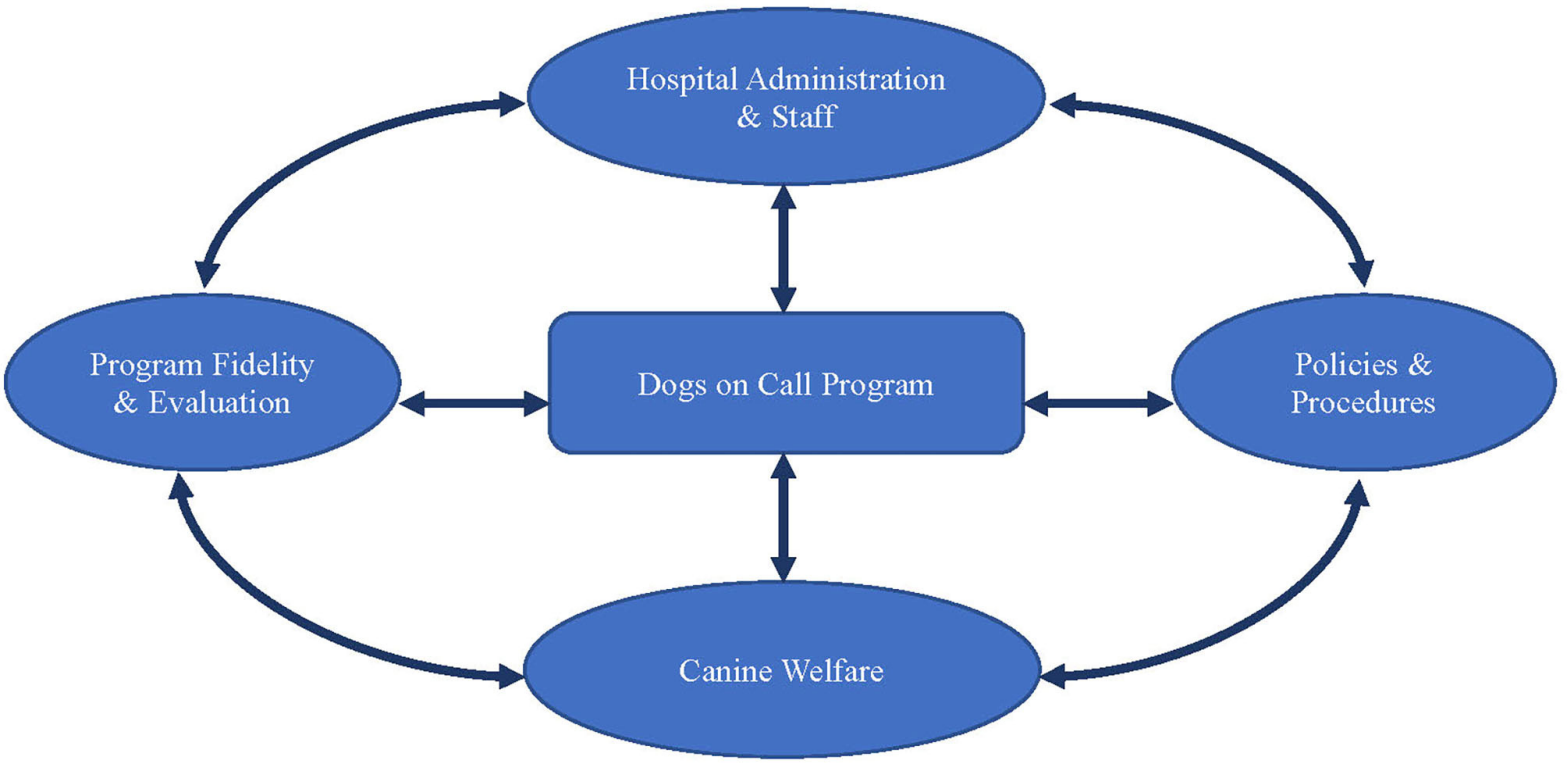
Integrated best practices program model—Dogs on call.

Dogs on Call is a therapy dog program established in the Center for Human–Animal Interaction at Virginia Commonwealth University (VCU) School of Medicine in 2001. The program model depicted in Figure 2 forms the basis for the following discussion. Dogs on Call provides CAI throughout the VCU Medical Center and meets the criteria for Best Practice:

- proposed as a standard suitable for widespread adoption (57) and selected as a model healthcare CAI program featured globally by Mission Critical Health
- published efficacy studies providing evidence base
- time-tested intervention operating in a major academic medical center since 2001
- established 19-year history of safety for humans and canines.

Dogs on Call policies and procedures were developed in concert with internal representatives from diverse disciplines including epidemiology, veterinary, and human medicine, and healthcare administration as well as informed by relevant professional guidelines related to infection control, animal-assisted interventions, and animal welfare. Policies and procedures are reviewed and updated on a regular basis to reflect changes in hospital policies and procedures, new knowledge from relevant fields, and results of formal and informal evaluation efforts. The program is manualized to promote standardized interventions delivered throughout the medical center.

Dogs on Call requirements include documentation of external therapy dog registration (Pet Partners or Alliance of Therapy Dogs), completion of VCU Medical Center volunteer services training, completion of Dogs on Call training, and adherence to manualized policies and procedures. The owner/handler and dog are evaluated and approved to participate together as a dyad in CAI programs. The unique bond and communication between them contribute to the safe practice of CAI and attention to canine welfare. Biannual meetings are held to provide program updates, reinforce program fidelity, provide continuing education, and solicit feedback. Teams are evaluated individually at least biennially to monitor program fidelity. Handlers participate in ongoing CAI related continuing education in such areas as canine behavior, patient safety, responding to difficult requests, and compassion fatigue. Program efficacy is documented through ongoing research and evaluation, disseminated through professional publications and internal and external education, and utilized in making program revisions. For detailed information on the Dogs on Call program see Barker et al. (57), and visit the program website^1^.

### Best Practice Recommendation: Involve Hospital Administration and Staff

Any novel program being considered by a hospital has a higher chance of being established, continued, and successful with administrative and employee involvement and support. Establishing such relationships and ongoing awareness of relevant research and practice are important foundations for any CAI program. For example, the involvement of medical, nursing, legal, volunteer services, veterinary, and other medical center administrators in developing Dogs on Call program policies and procedures were key to the program's credibility, acceptance, and ongoing support as a medical center program. One avenue for facilitating such involvement is establishing CAI executive and/or advisory committees involving key high-level administrators to build a sense of ownership and oversight by the hospital. Whether the CAI program is internal as is Dogs on Call, or external as with visiting community-based CAI programs, such committees serve to facilitate communication between the CAI program and hospital employees at all levels. Establishing liaison relationships with key hospital staff on services targeted for CAI promotes coordination and feedback regarding CAI activities. Liaisons can facilitate CAI scheduling, establish any needed service-specific orientation (e.g., pediatrics, psychiatry) for handlers, and provide CAI teams with unit-level assistance and support. Building relationships with frontline staff increases the likelihood of staff comfort with the CAI program and willingness to contact the program with any concerns, questions, or requests. These liaisons also enhance the CAI program's ability to monitor program implementation and resolve potential problems. Patient conditions in the hospital can change very quickly making real-time screening of patient appropriateness by front-line clinical staff critical to patient safety. Staff knowledge of the CAI provides an important framework for making informed decisions in screening patients and gauging the milieu for appropriateness for CAI on any given day.

### Best Practice Recommendation: Develop Informed Policies and Procedures

In addition to comprehensive knowledge of HAI and AAI, input from internal and external content experts representing other relevant disciplines is critical for developing policies and procedures to maximize therapy dog welfare and human safety in the hospital setting. At a minimum, representatives from human health (including epidemiology, medicine, and nursing), veterinary medicine, hospital risk management, hospital administration, and volunteer services should be involved. Their ongoing involvement provides a conduit for updating CAI policies and procedures based on evidence and regulatory changes from the diverse fields affecting a CAI program in a healthcare setting. For example, when COVID-19 cases began emerging in Virginia in March of 2020, Dog on Call operations were guided by the medical center's infection control department and hospital administration. Since Infection Control personnel and hospital leadership were knowledgeable of the Dogs on Call program, rapid infection control and visitation guidance was provided from an informed perspective of both Dogs on Call practices as well as COVID-19 risk to patients, CAI teams, and staff.

CAI policies and procedures are strengthened by incorporating relevant published guidelines. The publication of the Society for Healthcare Epidemiology of America guidelines for animals in health care facilities provides recommendations for service and therapy animals informed by science and current practice (52). A 2019 published manual for establishing and maintaining CAI in health care facilities represents another resource (57). Based on research and extensive experience, the manual includes recommendations on infection control reviewed by the Society for Healthcare Epidemiology of America.

It is important to note that at a minimum, most CAI programs require some type of handler-dog training and initial assessment to determine that minimum competencies are met. Therapy dog organizations providing such training and assessment vary in not only their initial requirements, but also requirements for renewal and their policies for members. Some renewals are payment-based with documentation of animal wellness while others require periodic re-evaluations of the dog/handler team. Such variability in requirements and policies highlight the need for hospitals to go beyond therapy dog organization requirements and develop comprehensive informed policies and procedures to maximize human and canine safety.

Policies and procedures should address CAI oversight as well. Identifying those responsible for the program establishes needed accountability and provides CAI handlers and hospital employees with individuals to contact with questions and concerns. In addition to monitoring therapy dog and handler ongoing compliance, oversight must include attention to canine welfare, and a system for monitoring where CAI is being conducted at any given time. This information will be needed in the event contact tracing is required. When a Dogs on Call handler thought his dog might have ringworm, he reported the incident to the hospital's Dogs on Call coordinator who immediately contacted Infection Control and contact tracing was initiated. Although the handler called later that day to confirm a veterinary consult ruled out ringworm (or any contagion), the contact system was able to be effectively initiated because CAI teams are monitored for time and location.

Oversight responsibility also includes addressing canine or handler problems or complaints that arise. Addressing any non-compliance with policies and procedures must be handled immediately for the safety of humans and canines and to ensure program integrity. For canine misbehavior, policies should detail clear steps to remediate the behavior (e.g., disruptive barking) or terminate program participation (e.g., any display of aggressive behavior). Oversight also includes implementing methods to acknowledge the contributions of CAI teams and to address ongoing recruitment and retention.

### Best Practice Recommendation: Develop Ongoing Program Fidelity and Evaluation Strategies

The most rigorous and well-informed CAI policies and procedures can only be effective if implemented as intended. Ongoing evaluation is an important component of any successful program to maintain program integrity and assess program worth and effectiveness (62). Dogs on Call monitors program fidelity by monitoring team members completion of annual hospital-mandated volunteer requirements, compliance with Dogs on Call program requirements, and completion of biennial evaluations of each team. Biennial evaluations consist of observing the team as they perform CAI in the hospital to monitor compliance with policies and procedures, observe handler-canine communication, and monitor canine body language for signs of fatigue or distress.

Hospital administrators are more inclined to support and continue programs that are shown to be effective. Providing evidence of efficacy involves evaluation. In addition to informing decision-makers, evaluations can provide valuable information for improving CAI processes, determining which patients benefit from CAI and in what ways, and identifying resource needs. For example, during the development of Dogs on Call, a needs assessment was electronically sent to all nursing coordinators in the hospital to determine interest in and concerns about a CAI program on their services. The results provided an indication of program demand, but just as importantly, identified areas for staff education on CAI to address their concerns. Ongoing studies of Dogs on Call have identified clinical populations benefiting from brief CAI (e.g., psychiatric, urology) (15, 30) as well as benefits for health care professionals (36). Such research also identified pediatric patient outcomes not significantly affected by CAI and unintended outcomes important for consideration in future research (e.g., low pediatric pretest distress and pain scores, use of pain medication) (25). Fidelity of implementation is critical for determining program reliability and efficacy. When delivery of CAI is not consistently conducted as intended, safety is jeopardized and results of any investigations are compromised.

### Best Practice Recommendation: Prioritize Canine Welfare Considerations

Although primary responsibility for canine welfare resides with the therapy dog owner, hospitals must also share some of that responsibility when CAI is approved on their premises. Hospital administrators are uniquely qualified to identify potential safety issues for canines in their facilities and develop strategies to minimize risk. For example, VCU Medical Center recognized the potential danger that dropped medication poses for therapy dogs. They proactively involved Dogs on Call representation on their medication safety subcommittee addressing this issue. Dogs on Call team orientation emphasizes continuous handler surveillance of the environment for potential risks for their dogs, including dropped medications, fluids, and food.

In addition to addressing risks, CAI programs must emphasize canine wellness in their policies and procedures as well. CAI programs benefit from involving hospital administrators in addressing therapy dog welfare. Such collaboration facilitates identifying adequate parking to accommodate safely unloading dogs, designating adequate exercise, rest, and elimination areas, identifying on-site or local veterinary resources for emergent issues, and developing processes for CAI team check-in and check-out to enable contact tracing, not only for possible canine-to-human zoonotic transmission but human-to-canine transmission as well.

In setting firm time limits for dogs in the hospital, the CAI program can address a canine welfare issue inconsistently addressed by therapy animal registration organizations (55). Setting firm time limits (e.g., 2 h maximum) for the dog on the premises provides a program regulation to support canine welfare and provide handlers with an objective rationale for denying requests to lengthen time in the hospital.

It is important to emphasize canine welfare in CAI orientation and continuing education, including understanding canine body language and recognizing when the dog does not enjoy the environment. Some dogs may not be comfortable in highly stimulating settings (e.g., pediatrics, emergency departments) but are very comfortable in more predictable surroundings (e.g., adult services, outpatient clinics). Handlers become very invested in participating in CAI and may not recognize changes in their dogs over time that may indicate they are fatigued or no longer enjoying the hospital setting. Periodic monitoring of the therapy dog team by CAI program staff can assist with identifying when a dog may need a break or retirement from CAI. Continuing education for handlers normalizing retirement can be helpful, but in the end the CAI program must be willing to administratively retire a therapy dog if it is in the best interest of the dog.

## Summary and Practical Implications

This paper began by summarizing the evidence supporting benefits of CAI in hospital settings while noting the existence of conflicting evidence and the need for further research. Several studies have documented positive results of CAI, but there is little consensus in the clinical populations studied, outcomes and measurements selected, and methodological rigor. The exceptions to these disparate positive findings are accumulated studies showing CAI benefits for cardiac and psychiatric patients.

To advance research on CAI in hospital settings, established CAI programs are needed. Yet the hospital setting presents unique challenges for the delivery of CAI. Sources of potential issues and challenges for humans and dogs involved in CAI in the hospital setting were presented based on the literature and the authors' extensive experience administering and conducting CAI. Best practice recommendations for CAI program processes and oversight were then provided with examples from a model best practice program operating in a major medical center.

### Practical Considerations

For people interested in establishing a CAI program in a hospital setting, gaining entry may seem like a daunting task. Identifying one or more key allies in the hospital can facilitate this process and provide valuable guidance on preparing a proposal that has a higher likelihood of being accepted by administrators. Hospital employees likely represent the surrounding community and their opinions can provide insight into views regarding pet ownership and the potentially favorable impact of a CAI program. However, it would be a mistake to assume that hospital employees are aware of hospital-based CAI and evidence supporting benefits. Our initial needs assessment at VCU Medical Center revealed many misconceptions and concerns about therapy dogs in the environment, including that dogs would be dirty, noisy, and disruptive in the environment and bring fleas, ticks, and other sources of illness to patients. An initial educational approach to introducing CAI may lay the groundwork for acceptance and support of the program.

Ultimately approval, support, and endorsement of CAI at the highest administrative levels is desired, but the process of securing such widespread support takes time, planning, and patience. The antecedent of Dogs on Call was a small pilot project conducted by the first author on inpatient psychiatry. Suggesting a pilot project as an entrée to CAI has advantages for those approving the project. “Pilot” implies temporary and therefore can be discontinued if the process and outcomes are not considered valuable or beneficial. Including an evaluation component can provide documented outcomes to support the program. Outcomes can include informal feedback from patients and staff and/or simple assessments measuring mood or anxiety. More than any other single factor, results documenting positive patient outcomes contributed to the growth in credibility and widespread support and institutional integration of Dogs on Call at VCU Medical Center.

Cost-effectiveness is a key consideration for hospitals considering any new program. A benefit of CAI is that therapy dogs reside with their owners in the community. Owners assume the costs of ownership including veterinary care, food, training, therapy dog registration, etc. They provide CAI as volunteers, incurring only a modest cost to the hospital for providing volunteer orientation and required training, vaccinations, and record-keeping. CAI programs need adequate funding to develop, implement, maintain, and evaluate the program. Identifying funding sources is challenging for CAI in any context with competing interests vying for a limited pool of financial resources. Successfully competing for funding against requests for healthcare and allied health personnel, equipment, patient needs, information technology, infrastructure and the myriad of other hospital needs is enhanced with evaluation data documenting the value of CAI in terms of benefits to patients, staff, and the organization.

Some programs, including Dogs on Call, establish themselves as non-profits eligible for tax-deductible donations and seek support from community foundations, grants, and individuals. Again, evaluation is key to demonstrate program efficacy to potential sponsors. Some CAI programs charge a nominal annual fee for membership and require members to purchase branded merchandise (shirts, dog vests) while others raise funds to provide membership and merchandise at no charge to members.

As the popularity of a CAI program grows, so does the demand for services. Recruiting, training, and monitoring additional CAI teams can strain existing program resources. Experiencing such demands, Dogs on Call developed a Leads Program to assist with these efforts. Experienced Dogs on Call members are recruited, educated, and trained to assist with recruiting and onboarding of new members and monitoring for program fidelity. Meeting regularly with Leads members enhances their program involvement, visibility, and recognition as senior level volunteers. Their experienced insights and feedback strengthen and inform program policies and procedures.

### Conclusion

Canine-assisted interventions (CAI) have the potential to complement traditional medical treatments in contributing to the health of hospitalized individuals. The hospital setting presents unique challenges to humans, dogs, and dog handlers in providing CAI effectively and safely. Recommended best practices are presented based on the literature and a model best practice program to guide hospitals and CAI programs in implementing programs that maximize canine welfare and human safety. More evidence of CAI efficacy with hospitalized patients is needed. To advance existing research, studies must be undertaken utilizing rigorous methodologies to investigate CAI programs that meet best practice criteria.

## Author Contributions

SB and NG conceived the idea for the paper together. SB provided the initial organization of the paper. All authors wrote the document in shared collaboration.

## Conflict of Interest

The authors declare that the research was conducted in the absence of any commercial or financial relationships that could be construed as a potential conflict of interest.

## Footnotes

^1^https://chai.vcu.edu/programs--services/dogs-on-call/
